# Chinese contemporary art teachers’ professional development in the 20th and 21st centuries within the multicultural framework

**DOI:** 10.1186/s40494-022-00692-8

**Published:** 2022-05-03

**Authors:** Jing Yang

**Affiliations:** Department of Fine Arts, Academy of Arts and Design, Qiqihar University, Qiqihar, China

**Keywords:** China, Contemporary art, Education, Online learning, Online platform, Pedagogy, Art

## Abstract

The study of contemporary art is one of the main means of implementing aesthetic education in China. This discipline allows a student to develop a system of spiritual values, reveal talents and acquire skills needed for professional growth. Chinese art is an integral part of the global cultural space, and Chinese contemporary art performs the functions of cultural reflection, occupying an important place in developing the state’s cultural identity. In the process of globalization, it is extremely important to study not only Chinese, but also world contemporary art, as well as the peculiarities of its teaching in the educational institutions of the PRC within the framework of the multiculturalism movement. The study objectives are to develop a unique online platform for teaching and learning contemporary art in China, to identify the features of art teacher training, to identify the methods of and trends in teaching contemporary art in the Chinese system of secondary and higher education, as well as to determine the most effective method of teaching contemporary art in China based on the experiment. In the course of the study, a plan and structure of the website containing audio and video materials, graphic editors and a conference system for students and teachers were developed. An innovative classification of the contemporary art movements was made in accordance with the tools and methods of creating art works. The styles of contemporary art were divided into groups: painting, sculpture/architecture, photography, cybernetic art, literature, optical art, theatrical art, mass culture, and hybrid art. The database was compiled by the masters of Art from China and Ireland, which emphasizes the internationality and multiculturalism of the project. An experiment was conducted to determine the level of effectiveness of different methods of teaching contemporary art in China. In the course of the experiment, the Spark platform developed for the study purpose was recognized as the most effective (56.1%). The effectiveness of the active method and passive method of teaching was 27.2% and 16.7%, respectively. The study results can be used in the study of various trends in contemporary art in the process of modernization and within the framework of pedagogical and commercial activities related to the work of exhibitions and galleries. In addition, they can be used to improve the methods of teaching contemporary art online and offline to the broad public audience and improve the professional skills of graphic or UI/UX designers, 3D modeling experts, engineers, editors, and IT developers. The practical significance of the developed online platform lies in the possibility of database use, practical application of the knowledge gained, and taking online lessons with Chinese and foreign teachers as part of a multicultural experience exchange.

## Introduction

Today China is one of the world leaders in the field of economy, which is mainly formed by the state. The Chinese authorities invest a considerable part of the budget in education, which can bring valuable personnel to the country. Thus, in 2020, China’s spending on education amounted to $831.3 billion [1]. Chinese education policy is driven by the need for literate, talented, out-of-the-box thinking people whose knowledge and skills can benefit the state, enhance, and strengthen its position on the global scale. The education system of the People’s Republic of China (PRC) is based on the principles of social justice while guaranteeing equal educational opportunities for all children [2]. In view of this, schoolchildren receive free education and students are allocated grants and scholarships. China willingly accepts foreign students expanding its socio-cultural boundaries and sharing experiences with other countries [3].

In the 1990s in China, 4% of citizens had higher education, the level of education and scientific research activity of the country was low and only tough legislative measures made it possible to get rid of the illiteracy epidemic [4]. As of 2021, the percentage of citizens with higher education was 15.5% [5].

The development of education has led to the technical and informational progress of China, which has been observed in recent decades. The expansion of space programs, breakthroughs in robotics and engineering, and the development of medicine and pharmacology have made China a world leader and raised the social and financial level of the population [6]. China has become an entity of international trade and politics, and one of the main exporters of technical and surgical equipment, nuclear reactors, television equipment, computers, etc. [7]. It is also one of the leaders in the global IT market. In 1998, the adoption of the Law on Higher Education of the People’s Republic of China made national educational institutions world-class universities staffed with high-class professors and innovative laboratories [8].

Higher education guarantees Chinese citizens decent wages and high social status; thus, studying hard and obtaining an in-demand specialization are the main goals of the Chinese [9]. Recognizing the importance of education is developed in people from childhood. Mass media are promoting the need to obtain higher education; this idea is cultivated in families, developed in kindergartens and schools. Primary and secondary education aims to prepare students for university developing their self-awareness and desire to continue education [10].

Today China is focused on increasing the rate of economic, social, and cultural growth in the face of high international competition, which necessitates training of highly qualified personnel in various fields [7]. Professional training in the context of constantly changing information flows, technological and cultural evolution requires an effective system for the preparation of competent teachers [11].

Pedagogical education in China is based on the ideas of Confucius and combines the traditional values of the Chinese nation with the modern values of the state and society and is focused on the world historical and pedagogical practice [2, 3]. Unlike traditional education, which focused on academic performance and ignored the value system and social development of an individual, the modern approach to education in the PRC is aimed primarily at building a moral, humane, and creative personality with extensive knowledge and the ability to apply it in practice [12]. One of the main trends in modern education in China is online learning, the implementation of online courses and conferences, webinars, and cyber lectures [13].

The study of contemporary art has a considerable impact on the development of spatial thinking, creativity, aesthetic worldview, and the ability to make practical decisions. This subject intends to familiarize Chinese students with alternative traditional types and styles of art, set up the process of forming the person’s vision of the world value system, and involve them in the international multicultural exchange [14].

Art has been one of the most important sectors of China’s cultural heritage since antiquity. In the middle of the 20th century, under the influence of socio-economic factors and external influences, cardinal changes took place in the art of China [15]. The cultural trends of Western Europe and Russia have influenced genre-thematic, stylistic, and technical segments of Chinese art; basic aesthetic principles also underwent a transformation. Chinese art has embarked on the path of forming its own identity, and various works of art get new plots and characters [16]. The emergence of Chinese contemporary art as a separate movement dates back to the end of the Cultural Revolution. The history of Western art, which became widely available during this period, inspired artists to experiment with changing artistic styles [17].

At the beginning of the 21st century, Chinese art acquired its own stable national traditions, overcame ideological pressure, began a partial autonomization of the creative process, and expanded the themes and variety of styles [18]. In the themes of the works, the focus of attention gradually shifted from social and ideological problems to personal-psychological and spiritual-moral ones [19].

Contemporary art in China is expressed in a balance between aesthetic heritage and modernization metamorphoses, while existing in a post-socialist context and post-traditional framework [20]. It demonstrates the clash of individual artistic freedom with Chinese government policies, Chinese traditions with modernization, Chinese identity with the art world within the framework of globalism [21].

Contemporary art is included in the curriculum of the Chinese school system; it is also included in the study of design, architecture, engineering, computer graphics, fine art photography, stage direction, journalism, psychology, and marketing [22]. One of the fundamental factors ensuring the effective process of teaching contemporary art in the PRC is the high level of methodological training of a teacher who is able to harmoniously organize the educational process to reveal and develop students’ talents, which results in the implementation of their creative ideas, the improvement of personalities, the formation of responsibility in the socio-cultural environment, as well as creative understanding and transformation of the real world diversity [23].

Chinese national culture has had a significant impact on the teaching of contemporary art. Evidence of this is the changes in the organization of educational activities in schools, where students were required to attend various public events related to the arts, such as museums of traditional and modern art, cultural sights, etc. A system of visits by contemporary cultural figures to schools was also established to give lectures and demonstrate their creativity; students’ volunteer work and performances in nursing homes in front of older people are also organized [24].

The peculiarity of teaching contemporary visual arts in China is the introduction of fresh bright ideas that allow students of different ages to master modern fine art techniques and express their inner worldview with the help of innovative technical tools, such as multimedia equipment, computer gadgets, engineering creativity tools, graphic design, and 3D modeling software [25]. Teaching methods that were mainly focused on the traditional and academic approaches have been greatly modernized in recent years. They ensured good theoretical knowledge but did little to develop a personal style and creative thinking of the person. Thus, the focus has been shifted to an innovative interactive network model of material presentation [11].

Modern art, although it has an avant-garde nature, is closely connected with the history of world culture and touches upon eternal themes. One of them is the study of human nature, the relationship between human and society, human and nature, human and God. At the same time, globalization and technical innovations provide new opportunities for the creativity of artists, give new visual resources, including digital ones [26].

Digital technologies make the learning process more personalized through access to real-time personal data, modern educational materials, web and mobile applications, archives, photo, video, and audio resources [27]. Thanks to innovative technologies, teachers have been able to create mixed learning environments, use digital tools for intermediate and final monitoring of study progress [28].

Technologies in the field of art teaching allow students to develop innovative abilities and creative thinking, help to learn the basics of modeling, graphics and creating various types of fine art using graphic editors, video editors and 3D design software [29]. Digital technologies for teaching art help prepare and transmit information in the process of teaching with interactive equipment. They promote visual perception of information, allow users to consider the details of various works of art, increase the motivation and transparency of the lesson. Technology makes it possible to demonstrate any artistic material to students using photo and video materials, graphic models, also allows them to visit museums and conduct excursions remotely [30].

Digital technologies open wide access to various information, including materials of interactive museums, intangible cultural heritage archives, online exhibitions, and galleries. Access to various objects of art in the digital space helps to broaden students’ knowledge breadth, helps them to become familiar with the understanding of various trends in contemporary art, contributes to the formation of aesthetic taste among students, allows them to form their own point of view, helps to get acquainted with the theory of art and the practice of creating various creative projects [31].

The rapid development of digital technologies has had a significant impact on the transformation of China’s contemporary art. Digital materials, technology, and innovation have expanded creative boundaries and opened up new artistic paths for creators [32]. The educational process in China is rapidly moving into the digital space - online surveys and tests replace oral surveys, homework is carried out on the basis of Internet resources, blogs, video hosting and social media platforms. Online learning management systems such as Moodle, ILIAS or Stud.IP are being introduced [33]. Distance learning is actively carried out using Zoom and Meet video conferencing programs, as well as modular platforms such as WebTutor [34].

The theory and practice of art education, built on the appeal to various types of contemporary art, the products of which are created in a virtual environment, is called digital arts pedagogy [35]. Digital arts are characterized by the virtuality of objects of artistic activity and its interactivity, which demonstrates a new educational area - the complication of actions while simplifying operations [36]. Thus, based on these characteristics of digital arts, teaching methods are built, such as:

- an integrated method that combines creative practice and the study of theory;
- methods aimed at familiarizing students with creative practice (reliance on a system of increasingly complex creative tasks, a method for explaining the sequence of actions and operations in a particular digital art, a method of author’s introspection);
- methods aimed at familiarizing with the knowledge necessary for the implementation of this activity (representation of theoretical material in a systematic form, the application of the rules for the interaction of expressive means, the method of applying figurative models of theoretical concepts, etc.);
- methods that stimulate students’ creative manifestations;
- methods of computer modeling of artistic and creative process [37].

In recent years, the problem of professional training of contemporary art teachers has become acute in China due to the development of the media field, in which new areas of art are rapidly emerging and developing, for example, Post-Internet Art, Brandalism, GPS-drawing. Contemporary art is not reduced to painting, sculpture and arts and crafts. It is developing and changing along with IT progress. Movements, such as Digital Art, video art, systems art, and media art, create the need to simulate and implement the learning process with the help of IT tools through online learning, which take into account both the characteristics of Chinese contemporary art and global trends [38].

In addition, the methodology of teaching contemporary art in China is not aimed at studying separate movements and their features but is generalized, which makes the professional and pedagogical activity of teachers superficial and less effective. Innovative training programs that are rather focused on separate contemporary art styles are inherent, to a greater extent, in individually designed courses, teacher training, or sector-specific specialties, but there is no comprehensive well-developed school and university curriculum in contrast to the traditional fine arts [7].

The professional competence of art teachers in China has its own distinctive features, which is due to the specifics of the mentality and trends in their perception of world traditional and contemporary art. In the 21st century, within the framework of the “soft power” policy and multiculturalism, the PRC authorities directed state resources to the development of art and contemporary art, in particular [39]. The policy of developing different areas of Chinese art and bringing it to the world level involves the assimilation of the pedagogical foundations of foreign and national educational traditions to form a new type of modern art teacher [8].

The development in China of cultural pluralism, the policy of equality of all members of society, regardless of ethnic, racial, gender and religious differences, became the basis for the emergence of a multicultural society in the country [40]. Multiculturalism is a policy of accepting the diversity and equality of different cultures. Multiculturalism is characterized by tolerance for cultural differences, the pursuit to protect the diversity of identities, the interests of various groups in society [36]. This policy strives for the diversity of society, since cultural exchange is a value of the modern world society within the framework of globalism [41].

In China, society has been a mix of many ethnic and cultural identities for several centuries, so multiculturalism in this country acts as a diversity of cultures within the same nationality [42]. Multiculturalism in modern China encourages social dynamism, which leads to the rapid development of the cultural sphere of the state [9]. It also helps to increase the creative potential of individuals and stimulates the development of contemporary art by blurring social boundaries, eradicating prejudice in society towards representatives of other cultures and ethnic groups, stimulates the development of critical thinking and pushes people to look for non-standard, innovative approaches to art [43].

In the mid-20th century, China pursued an active policy of cultural transmission and socialization of youth, while public education was designed to promote national unity and economic development of the country [44]. However, in the process of teacher training, there were no specialized courses on the features of multicultural education, methodological foundations for eradicating socio-economic, ethno-national and educational inequality in the student body [45]. Since the beginning of the 21st century, China has begun to prepare a new generation of educators who are trained in the specifics of multicultural education, who know the basics of teaching national unity, and who are able to prepare multicultural curricula [45].

The professional competence of a modern art teacher in China involves developed imagination, spatial thinking, the ability to create hybrid materials and compositions, knowledge of the basics of computer science and the latest graphic editors, as well as the adequate perception of art works related to various styles and movements of world and Chinese art [46]. The most important component of the competence of an art teacher in China today is the skills of professional and emotionally accurate perception and analysis of contemporary art works and movements [27].

In the process of teaching contemporary art, Chinese teachers try to focus on students’ artistic development rather than on the outline of the discipline basics. They help students comprehend the psycho-emotional, artistic, and aesthetic side of the world and national contemporary art [46].

The level of professional training of rising contemporary art teachers is determined by the formation and development of their compositional thinking. It is distinguished by revealing such student traits as independence, thinking originality, associativity, figurativeness, compositional meaning management, a sense of color harmony and disharmony, and visual memory [47].

This research is an original study of the characteristics of teaching contemporary art in China in a multicultural framework, as well as an attempt to develop a universal online platform for teaching contemporary art to schoolchildren and students in China. The media arena should be focused not only on theoretical knowledge but also on the creation of their own projects based on the knowledge gained about the world and Chinese contemporary art. The relevance of the study is determined by the rapid emergence of new contemporary art trends related to information technology, the modernization of the educational system in China, and the impact of globalization processes on it, as well as the poorly elaborated program for teaching contemporary art in the context of secondary and higher education on a large scale. Since today in China most modern art lessons are based on providing students with discreet theoretical knowledge, and the classical teaching didactics has little effect on students’ creative potential, it is necessary to develop an optimal innovative online platform that can systematize multidirectional trends in contemporary art. This can also help teachers improve their qualifications, expand knowledge, and give students of different ages and specialties not only knowledge about contemporary art in China and in the world, but also teach them practical skills for its application in creative and professional activities. Thus, students can get a deeper insight into the culture of humankind and the depths of the socio-cultural context of certain creative areas.

The scientific novelty of this research project lies in the development of a universal media arena for comprehensive teaching of contemporary art in China to students of all ages to expand their worldview and knowledge, involve them in the practical creative process within the network field, and improve the level of teachers’ qualifications and cultural exchange with foreign teachers through online lessons.

## Literature review

Features and trends of teaching contemporary art in China have been little studied. Most studies are aimed at studying the education system, which is due to the rapid growth of the economic and social level of the PRC, the emergence of new educational reforms and the globalization process that affects them [48]. The reason for the poor elaboration of the issue is the constant emergence of new trends in contemporary art and their focus on IT technologies [49]. Due to the emergence of new contemporary art movements, it is difficult to design an appropriate program that could completely cover the topic and to predict the impact of a certain trend on the development and professional qualities of students of different ages and specialties [50]. The issue of developing contemporary art pedagogy has also been little studied as most studies are focused on teaching fine arts. The modernization of the Chinese education system contributed to the adjustment of teacher education to the requirements of modern times and a change in the curricula, including plans for studying visual arts in the context of secondary and higher education systems. However, the conceptual foundations still rely on conservative guidelines to the detriment of innovative methods [51]. Training programs that raise the issue of contemporary art are mainly focused on visual trends while ignoring the digital direction, happenings, and process art [52]. Contemporary art is mostly taught to students of sector-specific specialties and is only partly found in secondary and high school curricula as part of the Art discipline, but not as an independent subject [53]. It is noted that contemporary art is difficult to teach as the diversity of trends requires the use of audiovisual tools and interactive practices in the course of the lesson [38]. In addition, the problem of visiting museums, exhibitions and interactive theaters arose in the context of the pandemic. Therefore, the study of contemporary art requires the expansion of the curriculum within the media space [54]. It is noted that the main trend in contemporary art teaching within the framework of Chinese secondary and higher education is the development of the emotional and figurative perception of the world, the rejection of a purely theoretical study of the subject and the concentration on its conceptual component. In other words, this refers to teaching students to independently form and express their personal concepts, as well as to create an original design solution using visual tools [25]. As part of studying contemporary art, students master visual activity through the use of integrated technologies, interactive learning methods, artistic and creative technologies, and IT solutions [8]. It is noted that China has been a pioneer in incorporating information and communication technologies (ICT4E) into the national education policy [29]. China is the leader in the application of new digital technologies in education. Chinese modern curriculum emphasizes course diversity and a multicultural teaching paradigm. Within the framework of art education and contemporary art teaching in particular, the curriculum combined the traditional Chinese educational model with digital technologies and innovative electronic resources [32]. Globalization processes and the contradictions related to them have set contemporary art education a task to prepare students for life in a multinational and multicultural environment, to develop their communication skills and cultural experience with the representatives of different nationalities [55]. Trends in contemporary art teaching in China aim to teach students and schoolchildren not only the peculiarities of national culture and art, but also the understanding of the diversity of world cultures, their value and uniqueness through multicultural education [3].

Some studies describe the features of teaching different contemporary art movements in China in a multicultural framework. For example, an extensive analysis of the impact of teaching digital art to high school students in China on the development of their creative abilities and the formation of social awareness was carried out [56]. In addition, the effectiveness of teaching media art to high school students and the influence of this factor on the choice of IT specialties to study at the university were described and analyzed [57]. It is noted that the possibility of network teaching of art expands the multicultural framework and promotes a cultural exchange of experience between students and teachers [58].

The diverse cultural environment in art education is a diversified trend in art and pedagogy. Art teachers in China help students understand traditional Chinese and world culture, as well as open their minds to new artistic elements [22]. The peculiarity of teaching contemporary art by Chinese teachers in the context of multiculturalism is manifested in applying the language of art and making the abstract content of contemporary art realistic. Thus, students move from the art form to the content while understanding the idea of a particular piece faster and more comprehensively [46].

Features of teaching contemporary art in different countries are found in foreign scientific articles. It is noted that most art schools in Europe are in the university system, and offer students standard bachelor’s, master’s and even Ph.D. degrees [45]. In European countries, the profession of an artist is considered not a craft, but an intellectual production, where theoretical disciplines are the key ones [28]. It is indicated that creative universities in such countries as Germany, Austria, Spain, Italy, and France receive comprehensive support from the state and private foundations; art education is free of charge at state universities, schools, and institutes [59]. It was noted that the recent trends in the integration of pedagogical science and its internationalization made it possible to study in more detail the features of pedagogical processes in various states [59]. It is pointed out that the teaching of art in most universities in Europe is free, which means greater independence of the teaching corps in determining the specialization of the university, the educational model, developing strategies and tactics for training teachers [59].

As for the art education of US teachers, the theory of education is the basic one, and the training is based on drawing with a pencil and paints, sculpture, installation, as well as separate courses on digital art and graphic design, practice in art studios, galleries, media sphere [60]. American higher art educational institutions include art practice, didactics and art history, museum pedagogy, and the psychology of art [60].

Russian scientific studies note that the interpretation of contemporary art cannot be taken outside of modern humanitarian methodologies [24]. For teaching contemporary art, case methods are offered, where the most valuable is work with specific situations, in the plane of which the work of various authors is placed, which can save teachers from attempts to exhaustively narrate art and from premature entry into the field of one or another theory or philosophy of art [30]. This approach is designed to help teachers fully reveal and demonstrate to students even the most provocative and extraordinary artistic practices that challenge the usual criteria for defining art [30]. It is noted that the case method allows one to proceed directly from the uniqueness and specificity of the artist’s style, and not from a strained idea of the tradition or direction of a particular type of art [24].

In the studies of South Korea, works of contemporary art and its various types are usually studied as creative strategies, which are the reaction of artists to certain world processes, such as historical events, transformations of public morality, social conflicts, natural disasters, psychological metamorphoses of individuals in society, etc. [44].

### Setting objectives

The research focuses on developing a unique online platform for contemporary art teachers and students of different ages with due regard to the international and Chinese cultural characteristics, as well as art movements. The data were collected and structured based on social media.

The study objectives were as follows:

- The development of a plan of an online platform that can improve the professional skills of Chinese teachers in the field of contemporary art within a multicultural framework, as well as increase the level of student involvement in the interactive learning process.
- Obtaining new experimental data on the effectiveness of the developed online platform in the context of teaching contemporary art in China based on the analysis of the statistical data obtained.

The main research tasks were as follows:

- To consider contemporary art as a way of moral and aesthetic development of individuals.
- To determine the features of teaching contemporary art in the Chinese system of higher education.
- To analyze the relationship between teaching contemporary art and students’ professional growth.
- To determine the influence of the developed online platform for studying contemporary art on developing students’ professional identification.

## Methods and materials

### Research design

The research methodology is represented by design and experiment methods [43]. At the first stage of the experiment, the design method was used in the development of the program and online platform with the help of digital analysis and visualization tools. In the second stage, the method of the experiment was presented in online and offline formats according to the developed program. In the course of the study, a plan of an online platform (Spark) for studying and teaching contemporary art by Chinese schoolchildren, students of all specialties and art teachers was developed. Based on the developed media platform, an experiment was conducted in order to evaluate the effectiveness of this information and interactive tool for teaching contemporary art in China.

The study objects were as follows:

- high school students of Western Academy of Beijing, Beijing Chen Jing Lun High School, and Beijing No. 35 High School (Beijing) aged 16–17;
- students of various specialties of Beijing Institute of Technology, Renmin University of China, and Communication University of China (Beijing) aged 18–20;
- students of various specialties of Beijing Film Academy, Tsinghua Academy of Arts & Design, and the Central Academy of Drama (Beijing) aged 18–20;
- postgraduate students of Beijing Normal University, Beijing Academy of Educational Sciences, and Peking University who practice teaching within the Art History discipline aged 24–25.

The study subjects were:

- 4 teachers from the Central Academy of Fine Arts who conducted online lessons in the study groups;
- 2 teachers from the Chinese Academy of Sciences who conducted face-to-face lessons in the study groups;
- 2 independent art experts from the China Academy of Art who administered the final exam of the respondents;
- 2 programmers from the UX design agency Henan Cloud Dream Internet Technology Co., Ltd. (Zhengzhou, Henan) who developed the backbone of the website and mobile application;
- Masters of Arts from the Power Station of Art Museum of Contemporary Art (Shanghai);
- 2 Masters of Arts from the UCCA Center for Contemporary Art, the UCCA Foundation group (Beijing);
- 1 PhD in Arts from the CCADerry Center for Contemporary Art (Londonderry, Ireland).

Art critics compiled a database of subjects and performances of contemporary art on a volunteer basis; the conferences were held through social media. The online platform plan was developed to improve the process of contemporary art teaching in China and optimize contemporary art programs in secondary and higher education systems of China.

### Sample study

The selection of respondents was based on the simple random sampling method. There were no special criteria in the selection of experiment participants, in addition, one of the important conditions for participation in the experiment was the study of contemporary art in China. As a general population, students of Beijing schools and universities aged 16 to 25 were considered. Thus, 3 secondary schools, 3 universities, 3 universities of arts, and 3 pedagogical universities in Beijing were randomly selected; 15 people were randomly selected from each educational institution.

A total of 180 respondents aged 16–25, male (50%) and female (50%), took part in the experiment.

The sample is relevant as it corresponds to the research objectives. The respondents were students of different educational institutions, different specialties, and ages, so the results of the experiment can be applied to the general population.

### Features of the intervention

The experiment was based on online group lessons and lasted 110 days (from 2021-07-10 to 2021-10-27).

The statistical error of the experiment results does not exceed 2.7%.

The respondents were divided into 4 groups of 45 people each. Each group studied the Contemporary Art discipline in accordance with the various teaching methods determined by the age and specialty:

- Group A involved schoolchildren aged 16–17 years.
  Group A respondents were divided into 3 subgroups of 15 people. Subgroup A1 from the Western Academy of Beijing studied Contemporary Art based on passive learning (lectures with handouts) in the university classroom; subgroup A2 from Beijing Chen Jing Lun High School studied it in person based on the active learning method (discussion with the use of audiovisual materials); A3 subgroup from Beijing No. 35 High School studied the subject online based on the developed media platform.
- Group B - university students aged 18–20 years.
  Group B respondents were divided into 3 subgroups of 15 people. Subgroup B1 from Beijing Institute of Technology studied Contemporary Art based on the passive method of learning in the university classroom; subgroup B2 from Renmin University of China studied it in person based on the multimedia method; subgroup B3 from Communication University of China studied the discipline based on the developed media platform.
- Group C - art university students aged 18–20 years.
  Group C respondents were divided into 3 subgroups of 15 people. Subgroup C1 from Beijing Film Academy studied the subject based on the passive learning method; subgroup C2 from Tsinghua Academy of Arts and Design relied on the multimedia method to study the discipline; subgroup C3 from the Central Academy of Drama used the developed media platform.
- Group D - graduate students of pedagogical specialties aged 24–25 years.
  Group D respondents were divided into 3 subgroups of 15 people. Subgroup D1 from Beijing Normal University studied the subject in accordance with the passive method of learning; subgroup D2 from Beijing Academy of Educational Sciences relied on the multimedia method; subgroup D3 from Peking University studied the discipline on the developed media platform.

Classroom learning took place at the Chinese Academy of Sciences (Beijing).

### The study was divided into 3 stages

At the first stage, the online platform was implemented, which involved the development of the structure and backbone of the online platform, data collection and grouping within 30 days. At this stage, the selected visual materials were synchronized with the data of the Open Media Library and the YouTube video platform. The website interface was prepared in Chinese and English. The HTML code of the online platform was also prepared, and the selected data were analyzed.

At the first stage, the structure of the online platform was developed with the help of digital analysis and visualization tools with the support of the YouTube video platform and the 3DMax graphics software.

The platform has been designed for the convenience of grouping information and visualizing data by contemporary art movements. The developed program is based on the study of various areas of contemporary Chinese art: Painting, Sculpture/Architecture, Photography, Cybernetic Art, Literature, Optical Art, Theatrical Art, Mass Culture Art, Hybrid Art. The developed program aimed at art history (theoretical knowledge of various contemporary Chinese art types), creativity skills (the ability to create their own works of art), and axiological skills (skills development to adequately evaluate works of art).

The project aims to solve the following problems:

- To guarantee everyone open access to information about various world and Chinese contemporary art movements.
- To provide art teachers and students with access to graphic programs of separate areas of media art.
- To optimize the teaching process in the format of online conferences with Chinese and foreign teachers.

The contemporary art movements were divided into 9 groups in accordance with the method of art work creation:

- Group A: Painting.
- Group B: Sculpture/Architecture.
- Group C: Photography.
- Group D: Cybernetic Art.
- Group E: Literature.
- Group F: Optical Art.
- Group G: Theatrical Art.
- Group H: Mass Culture Art.
- Group I: Hybrid Art.

Also, the movements of contemporary art were divided into 7 subgroups in accordance with the timeframe:

- 1950s.
- 1960s.
- 1970s.
- 1980s.
- 1990s.
- 2000s.
- 2010s.

In addition, they were divided into five groups according to the place of origin:

- USA/Canada/Australia.
- Latin America.
- Europe.
- Africa.
- Asia.
- China.

A system of grouping various contemporary art styles by the methods of creating works of art was formed in order to facilitate the learning process. Contemporary art movements have been divided into:

*Painting.* This section included such movements as Abstract Expressionism, American Figurative Expressionism, Regionalism, Bay Area Figurative Painting, COBRA, New York Figurative Expressionism, Action Painting, Modular Art, Nuclear Art, Composite Painting, Chicano, Hard Edge Painting, Abstract illusionism, Street art, Artivism, Brandalism, Nanyang style, etc. These styles show the realization of the artist’s personal creative potential through painting.

*Sculpture/Architecture.* This section includes such areas as Geometry of Fear, Modular Constructivism, New Front of Arts, Mid-Century Modern, Kinetic Art, Plop Art, Gas Sculptures, Installation, Renewable Energy Sculpture, etc. These movements have reached a new level and skillfully demonstrated both massive forms and kinetic sculptures.

*Photography.* This section includes such movements as Lenticular Printing, Copy Art, Mec Art, Photorealism, Fax Art, the Red Shirt School of Photography, etc. These movements reflect the beauty and ugliness of the world through the prism of copiers, printing units or cameras.

*Cybernetic art.* This section includes Glitch Art, Media Art, Algorithmic Art, Procedural Art, Systems Art, Video Art, Information Art, Net Art, Virtual Art, Art House Game, Post-Internet Art, etc. The works of art in these areas are created with the help of both computer technology and graphic design software.

*Literature.* This section includes Art and Language, New Slovenian Art, Artist’s Book, Graffiti, Mission School, Signalism, etc. These movements are the actual forms of the artistic self-expression of the creator; at the same time, they show the work and express its value through literary devices.

*Optical art.* This section includes Light and Space, Holography, GRAV and others. These movements are focused on the perception of light, volume, and scale. The works are created with the use of glass, neon, and fluorescent lamps.

*Theatrical art.* This section involves Actionism, Interactive Art, Performance, Gutai, Art of Endurance, Happening, Process Art, etc. These movements aim to blur the lines between art and reality making the individual not only the creator of a work of art but also its direct part.

*Mass culture art.* It involves Appropriation, Trash Art, Artist’s Multiple, Capitalist Realism, Narrative Figuration, Pop Art, Culture Jamming, Neo-Pop, Mass Surrealism, Food Art, Deviant Art, etc. These movements depict or use consumer products, brochures, and images of mass culture as creative tools.

*Hybrid art.* This section includes such movements as Combined Painting, Shock art, Auto-destructive Art, Psychedelic Art, Neodada, Fluxus, etc. These movements involve the introduction of different techniques and trends into the work while combining them in an unusual manner. For example, painting can be combined with sculpture, technology, biomaterials, computer graphics, and interactive performance.

**Fig. 1:**
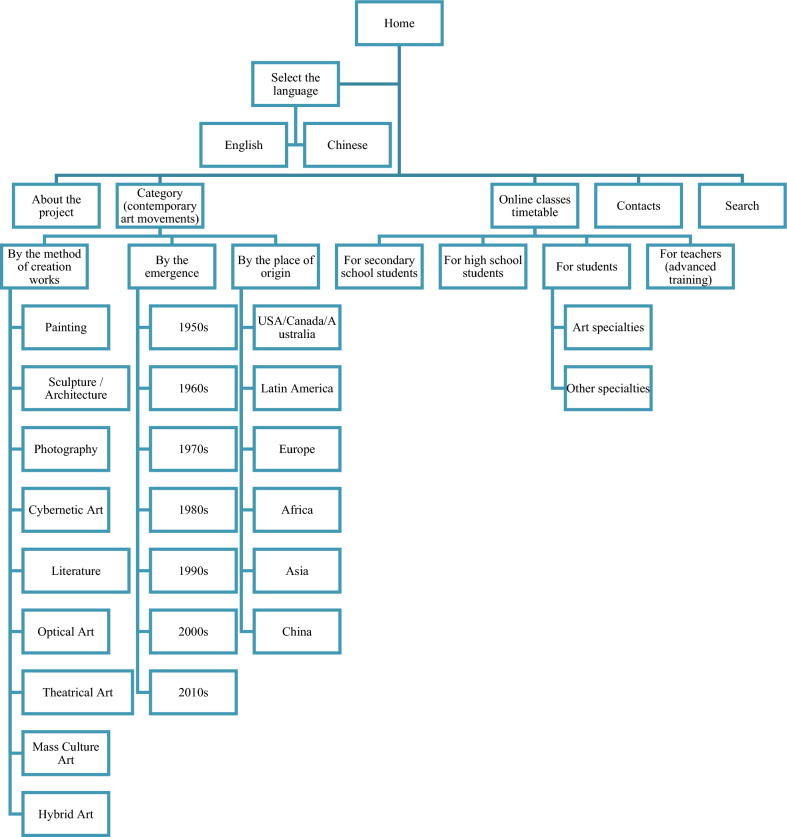
The online platform for studying and teaching contemporary art in China

Based on the classification of contemporary art movements, the structure of an online platform for studying and teaching contemporary art in China was developed (Fig. 1).

At the second stage of the experiment, online and offline classes were held. There were four 1.5-hour lessons a week for 70 days. The third stage was a follow-up stage, which involved the analysis and assessment of the knowledge and moral growth of the respondents by independent experts. The second stage lasted 10 days.

### Statistical analysis

The effectiveness of the methods under study was evaluated on the basis of the Bloom’s Taxonomy model [61].

The effectiveness of different teaching methods was analyzed with the help of the examination test compiled by independent experts based on the Bloom’s Taxonomy model.

The mathematical and statistical analysis was performed in SPSS-12.0.

The general assessment of the dynamics of moral changes and the level of knowledge was made on the basis of statistical results, reports prepared by the teachers of each group and independent expert teachers who did not participate in the first and second stages of the study.

### Research limitations

The limitations of the study are due to the possible inaccuracy of the data processing methodology as it is partly based on the personal observations of the teachers. To some degree, the analysis of the effectiveness of different teaching methods was based on the subjective opinion of the teacher of each subgroup.

The most difficult task was to achieve accuracy in the analysis of practical activities as their interpretation to a large extent depended on the teacher rather than on specific results.

In addition, the study limitations are associated with the possible traffic overload of the online platform due to the pandemic and remote learning, as well as with the obstacles that the platform may face at the legislative level when registering qualified teachers. The adoption of the Spark platform as part of China’s educational programs may lead to the need to change the curricula not only of art teachers but the curriculum of the educational institution.

### Ethical research issues

To minimize inaccuracies, independent experts analyzed the theoretical and practical examination papers anonymously. That is, the experts did not know which subgroup the respondent belonged to, and the students’ works were assigned numbers.

## Results

In accordance with the established goals, the structure of a universal online platform for teaching contemporary art in schools and universities of China was developed, and the effectiveness of this media platform was determined in the context of its comparison with other methods of teaching contemporary art.

Based on the model for assessing the effectiveness of student learning (Bloom’s Taxonomy Model) proposed by Benjamin Bloom [61], in the course of a controlled study, a comparative analysis of the main methods of teaching art and the developed online platform (Spark) was carried out to determine the level of its effectiveness.

The effectiveness of student learning was assessed based on the following 3 criteria:

1. Knowledge—the level of respondents’ cognitive development.
2. Attitudes—the level of respondents’ emotional development.
3. Skills—the level of respondents’ psychomotor development.

The criteria for evaluating student learning effectiveness are presented in Bloom’s Taxonomy and imply that the level of students’ cognitive development demonstrates the ability to memorize and reproduce the studied material and knowledge of specific facts. The level of students’ emotional development demonstrates the ability of individuals to transform material from one form of expression to another, that is, to interpret the material. The level of psychomotor development of students demonstrates the ability to use the studied material in specific conditions and new situations [61].

Based on the observations of the teacher and an independent pedagogical expert group, as well as the mathematical and statistical analysis of the examination work as part of the analysis of Group A, it was revealed that the Spark platform, which was used to train subgroup A3, has turned to be the most effective in accordance with the first item of the Bloom’s criterion (item 1)—53.4%. It was followed by the active learning method used in subgroup A2 (33.3%) and the passive learning method used in subgroup A1 (13.3%).

According to item 2, the active method of learning was in the first place in terms of efficiency (42.2%) followed by the Spark platform (37.8%) and the passive method (20%).

According to item 3, the Spark platform showed the highest efficiency (60%) followed by the active learning method (26.7%) and the passive learning method (13.3%).

**Fig. 2:**
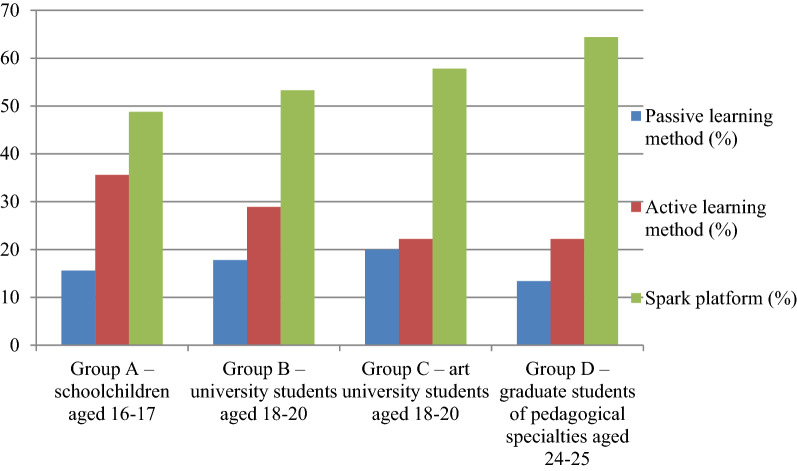
The effectiveness of different methods of teaching contemporary art in the groups under analysis

Thus, the Spark platform was recognized as the most effective method of teaching contemporary art (48.8%) to high school students within the framework of multiculturalism. The effectiveness of the active and passive methods of learning was 35.6% and 15.6%, respectively (Fig. 2).

As part of the analysis of Group B, it was revealed that the Spark platform, which was used to train subgroup B3, has turned to be the most effective in accordance with the first item of the Bloom’s criterion (item 1)—46.7%. It was followed by the active learning method (subgroup B2)—33.3%, and the passive learning method (subgroup B1)—20%.

According to item 2, the Spark platform was in first place in terms of efficiency (53.4%) followed by the active method of learning (33.3%) and the passive method (13.3%).

According to item 3, the Spark platform demonstrated the highest efficiency—60%, followed by the active and passive learning methods—20% each.

The Spark platform was identified as the most effective method of teaching contemporary art (53.3%) to students of multidisciplinary universities. The effectiveness of the active and passive methods of learning was 28.9% and 17.8%, respectively (Fig. 2).

Group C analysis:

According to item 1, the Spark platform (subgroup C3) ranked first in terms of effectiveness (66.7%). It was followed by the active method of learning (subgroup C2, 20%) and the passive learning method (subgroup C, 13.3%).

According to item 2, the Spark platform is the most effective (46.7%); it is followed by the passive learning (40%) and active learning (13.3%) methods.

According to item 3, the first place is taken by the online platform “Spark” (60%), the second - the active learning method (33.3%), and the third - the passive learning method (6.7%).

The Spark platform was identified as the most effective method of teaching contemporary art (57.8%) to art university students. The effectiveness of the active and passive methods of learning was 22.2% and 20%, respectively (Fig. 2).

Group D analysis:

According to item 1, the Spark platform (subgroup D3) ranked first in terms of effectiveness (73.3%). It was followed by the active method of learning (subgroup D2, 20%) and the passive learning method (subgroup D1, 6.7%).

According to item 2, the Spark platform is the most effective (53.3%); it is followed by the passive learning (26.7%) and active learning (20%) methods.

According to item 3, the Spark platform is the most effective (66.7%); it is followed by the passive learning (20%) and active learning (13.3%) methods.

The Spark platform was identified as the most effective method of teaching contemporary art (64.4%) to art university students. The effectiveness of the active and passive methods of learning was 22.2% and 13.4%, respectively (Fig. 2).

**Fig. 3:**
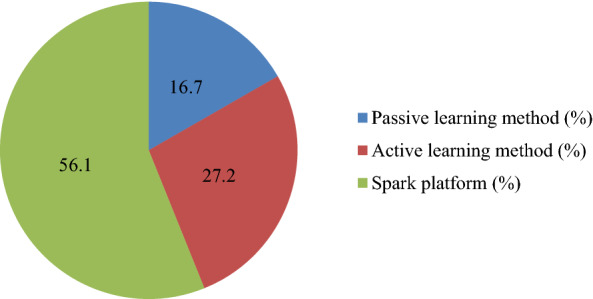
Assessing the effectiveness of contemporary art teaching methods in a multicultural framework

Thus, as part of the experiment, the Spark platform was identified as the most effective method of teaching contemporary art (56.1%) to schoolchildren and students of various specialties. The effectiveness of the active learning and passive learning methods was 27.2% and 16.7%, respectively (Fig. 3).

## Discussion

In this study, the effectiveness of several most popular methods of teaching contemporary art was analyzed and compared with the Spark platform developed as part of this project. In view of the reform and modernization of the education system of China, as well as the changeable dynamics of the information technology orientation of modern Chinese pedagogy, the features and methodology of teaching contemporary art in schools and universities of China have been only partially considered in foreign studies.

For example, US studies noted that contemporary art education in China is developed on the ideological basis of Confucianism, classical aesthetics, and Taoism [14]. The curricula cover the biographies of famous artists and the history of the emergence and development of major contemporary art movements and draw attention to the development of the Chinese contemporary art market [38]. Many sources have raised the issue of the impact of globalization on artists and the artistic mood of contemporary art in China [48]. The limitations of teaching this subject outside narrowly focused courses and the presence of organizational problems that significantly affect the quality of training of contemporary art teachers in the PRC were noted [50].

Features of teaching contemporary art in China have also been considered in European countries. Studies have noted that at the end of the 20th century, China adopted the policy of reforms and publicity while reorienting the curricula of schools and universities towards the value development of students and focusing on teaching art to cultivate a creative personality [14]. However, there were some system imperfections which focused on the study of traditional Chinese and world arts while paying little attention to modern trends [62]. It was highlighted that modern art is not given due attention in Chinese schools due to the specifics of certain areas. Chinese traditional art was based on creation and aesthetics, as well as bringing beauty to society. Destructive tendencies, nihilistic and hedonistic moods can be traced to a large extent in contemporary art. These can have a considerable impact on the worldview of students. Thus, the education system of the PRC imposes unofficial censorship on most areas of contemporary art allowing them to be studied only superficially in the context of art history or excludes them from the curriculum at all [46].

In Russia, no research has been conducted on teaching contemporary art in China, but the education system of the modern PRC, as well as the methods of teaching fine arts in schools and art and pedagogical universities, have been analyzed [11]. Russian studies note that in recent years, art education has been rapidly developing in Chinese educational institutions. In all provinces, appropriate administrative units and municipalities with full-time employees responsible for the effectiveness of teaching this subject and modernization of programs in accordance with the globalization processes and the needs of the time have been created [63].

In China, the issue of teaching contemporary art is raised quite often. The research of Chinese scientists is aimed primarily at studying the issue of teaching this subject in specialized art and pedagogical universities. It is noted that contemporary art passionately explores all possible tools of creation and expression, from digital technologies to the human body. It is constantly developing and going beyond the limits. Thus, a more in-depth study of art will allow students to learn to think critically and look for the new ways of self-expression [49, 64]. This will not only increase the intelligence level of individuals and expand their worldview, but also encourage creative thinking while traditional art education, which is solely focused on creating beautiful paintings, will help to express the talents of only certain students without providing a platform for alternative ways of self-development for everyone [16].

The role of the passive method of learning art was considered in Canadian studies, which confirmed the low effectiveness of this method in shaping the worldview, value orientations, aesthetic taste, and multicultural sentiments among students [65]. In another research work, the low effectiveness of the passive learning method in terms of involving students in the educational process was noted. It does not develop the creative aspect of an individual, does not allow them to fully transfer their personal experience to the visual plane due to the theorization of the process [65].

The results of the above studies are similar to the data obtained in the present study during the experiment, as the effectiveness of the passive method of learning contemporary art was only 16.7% of the other methods under study.

The effectiveness of the active method of learning art is noted by the 2019 data described in the Journal of Physics Conference Series. However, the study indicates that this method is effective only in the study of classical and traditional art but is inferior in effectiveness to interactive technological methods in the study of contemporary art due to the wide variety of its movements and the digital orientation of many of them [66].

The present study author agrees with this conclusion as the experiment conducted in this study showed the average effectiveness level of the active contemporary art learning method (27.2%) in comparison with other learning types.

As of 2021, the coronavirus (COVID-19) pandemic has become not only a medical, but also a social crisis forcing educational institutions to switch to online distance learning [27]. However, various studies note that online learning has become popular now not only because of the quarantine measures, but also due to the fact that technological development requires the adoption of new learning methods as in some cases traditional face-to-face lessons are ineffective. Contemporary art has many movements based on information technology that require appropriate study through online lessons and the involvement of digital arenas [67]. Online learning has been recognized as one of the most effective methods of teaching traditional and contemporary art within the framework of multiculturalism due to its interactivity, information, and technology features [19, 68].

The data of the above studies confirm the results obtained in the present study. Among all the methods analyzed, the Spark platform turned out to be the most effective (56.1%) in the context of teaching contemporary art to schoolchildren and students of various specialties.

In a joint study of Russia and the Netherlands, an analysis of distance learning effectiveness was carried out as part of an online course conducted by a teacher from the Netherlands for students of the Economics Department of the Chelyabinsk State University (ChelSU) with the aim of obtaining distance learning experience in an international educational environment and acquiring knowledge on the topic “Supply Chain Finance” (SCF). The teaching material of the course was presented by lectures recorded by the teacher and available online. A combination of lectures and online workshops held to discuss homework allowed students to gain a deeper understanding of the SCF topic. Bloom’s Taxonomy was used to evaluate the effectiveness of student learning [69, 70], as in the present study. The learning objectives of the online course presented in the Dutch-Russian study were formed in accordance with Bloom’s taxonomy, which includes three levels of learning: knowledge, attitudes, skills [71, 72]. In the present study, the same criteria for evaluating the effectiveness of student learning were applied for the online platform “Spark” developed during the research.

In this study, the author has developed a plan for an online platform for teaching contemporary art to a wide audience. In China, there are media arenas of similar themes in the format of courses and online lessons (An Introduction to Modern and Contemporary Art, Art and Tech courses, Media Art and Creative Industry Services), as well as online galleries (Capsule Shanghai, Galerie Urs Meile, de Sarthe), but they provide services on a paid basis as webinars for retraining or advanced training and do not fully cover all the movements of contemporary art in the world and China.

### Conclusions

In this study, a plan for an online platform for teaching contemporary art in China in a multicultural framework was developed. In addition, a comparative analysis of the developed platform with other methods of teaching art was carried out. An experiment to determine the most effective method of teaching contemporary art in China within the framework of multiculturalism was conducted. The mathematical and statistical analysis of the data obtained made it possible to determine the Spark platform developed within the framework of this project as the most effective method of teaching contemporary art to schoolchildren, students, and graduate students of various specialties (56.1%). The features of teaching fine arts in schools and universities of the PRC were identified, and the place of contemporary art as an academic subject in the educational programs of schools and universities was analyzed. Positive trends in the formation of worldview and critical thinking, as well as the development of creativity in the study of contemporary art, were noted.

The creation of a new media platform capable of covering all movements of the world and Chinese contemporary art will contribute to students’ abilities development due to the diverse and multidirectional aspects. It will help teachers improve their skills, and ordinary people will get acquainted with the world of art of the 20th-21st centuries and broaden their horizons. It will provide an opportunity to influence the consciousness of art education in China through the modernization of curricula. It will open up prospects for improving the efficiency of pedagogical activity outside of highly specialized educational courses. It will improve the quality of remote learning in the context of the pandemic. The study revealed the features of teaching contemporary art in China in various online and offline educational institutions as an independent subject and part of the art education program. Also, the media platform developed during the study can be used in teaching art in other countries, as it offers English-language functionality and has opened archive that allows users to add own artworks to the personal account, as well as suggest topics for discussion. Studying the works of Chinese contemporary art will allow foreign users to learn the language and history of China and better understand the Chinese mentality, become spiritually closer to the inhabitants of other countries, which can contribute to the development of multiculturalism in today’s global society.

The study results can be used in the art history practice to study contemporary art in person or interactively, as well as in the field of pedagogy to study the methods and tools for teaching students of different ages a certain type of art. In addition, the findings can be used in political activities to increase the country’s authority on the world stage and to present the contribution of national artists to the world culture. The study is universal as it can find its place in Chinese and world scientific, cultural, pedagogical, and social activities.

## Data Availability

Data will be available on request.
